# Lactate Clearance Predicts Survival Among Patients in the Emergency Department with Severe Sepsis

**DOI:** 10.5811/westjem.2015.10.27577

**Published:** 2015-12-08

**Authors:** Sundeep R. Bhat, Kai E. Swenson, Melissa W. Francis, Charles R. Wira

**Affiliations:** * Kaiser Permanente Santa Clara Medical Center, Department of Emergency Medicine, Santa Clara, California; † Uniformed Services Residency in Obstetrics and Gynecology, National Naval Medical Center, Bethesda, Maryland; ‡ Yale School of Medicine, Department of Emergency Medicine, New Haven, Connecticut

## Abstract

**Introduction:**

Lactate clearance has been implicated as a predictor of mortality among emergency department (ED) patients with severe sepsis or septic shock. We aimed to validate prior studies showing that lactate clearance during the ED stay is associated with decreased mortality.

**Methods:**

Retrospective dual-centered cross-sectional study using patients identified in the Yale-New Haven Hospital Emergency Medicine sepsis registry with severe sepsis or septic shock who had initial lactate levels measured in the ED and upon arrival (<24 hours) to the hospital floor. Lactate clearance was calculated as percent of serum lactate change from ED to floor measurement. We compared mortality and hospital interventions between patients who cleared lactate and those who did not.

**Results:**

207 patients (110 male; 63.17±17.9 years) were included. Two reviewers extracted data with 95% agreement. One hundred thirty-six patients (65.7%) had severe sepsis and 71 patients (34.3%) had septic shock. There were 171 patients in the clearance group and 36 patients in the non-clearance group. The 28-day mortality rates were 15.2% in the lactate clearance group and 36.1% in the non-clearance group (p<0.01). Vasopressor support was initiated more often in the non-clearance group (61.1%) than in the clearance group (36.8%, p<0.01) and mechanical ventilation was used in 66.7% of the non-clearance group and 36.3% of the clearance group (p=0.001).

**Conclusion:**

Patients who do not clear their lactate in the ED have significantly higher mortality than those with decreasing lactate levels. Our results are confirmatory of other literature supporting that lactate clearance may be used to stratify mortality-risk among patients with severe sepsis or septic shock.

## INTRODUCTION

The incidence of severe sepsis in the United States has increased steadily over the past two decades, with an estimated 3.1% of all patients who present with infection to the emergency department (ED) meeting criteria for severe sepsis.^1^ Over half of all septic patients require intensive care unit (ICU) admission,^2^ and among those with organ dysfunction, overall mortality rate may approach up to 70 percent.^2^–^4^ The advent of protocolized care has ushered in an era of ED outcomes research among patients with severe sepsis and septic shock with an objective of optimizing physiologic derangements occurring during the sepsis cascade. Direct mortality benefits have been shown in several studies.^4^–^6^ However, there are a limited set of methods to risk-stratify patients with severe sepsis and septic shock,^7^–^11^ and current tools require complex sets of variables or invasive monitoring, which may not be immediately available in the ED setting.^12^–^14^ There similarly remains a need for non-invasive endpoints to resuscitation that could tell providers they are successfully reversing physiologic derangements.

Serum lactic acid levels have long been identified as a diagnostic tool for global tissue hypoxia and therefore can serve in identifying patients with severe sepsis;^15^–^17^ however, in recent years there has also been recognition of the prognostic value of serum lactate measurement.^18^–^20^ Increased initial lactate values have been associated with mortality among all-comers with sepsis,^20^–^22^ as well as specifically in ED patients with sepsis.^19^ Based upon these findings, international sepsis guidelines now suggest routine measurement of lactate among patients with severe sepsis and immediate resuscitation for septic patients whose serum lactate measurement is greater than 4 mmol/L.^23^

Relatively few studies, however, have examined the role of lactate clearance or serial lactate measurements as endpoints among patients with severe sepsis or septic shock patients presenting in the ED.^18^,^24^–^26^ Several hospitals are now incorporating sepsis bundles into standard practice, and these bundles often suggest repeat lactate measurement,^27^–^29^ yet there are limited data available to show what clinical significance the serial measurement has. Our aim was to evaluate the predictive value of lactate clearance on 28-day in-hospital mortality and to investigate secondary outcomes such as need for particular treatments and interventions. We hypothesized that patients with severe sepsis or septic shock who present to the ED and have evidence of lactate clearance upon admission to the hospital would have lower in-hospital mortality rates than those who did not clear initial lactate levels.

## METHODS

The study was performed at a dual-site teaching hospital ED with nearly 100,000 patient visits annually. It was a retrospective cross-sectional study using patients identified prospectively in the Yale-New Haven Hospital Emergency Medicine registry. The study was approved by the Yale Human Investigation Committee for the review of medical records by study personnel. The registry is comprised of a patient list created between July 1, 2005, and July 31, 2008. In a systematic and standardized fashion, we prospectively and consecutively identified sepsis registry patients during predefined time periods at two EDs as a quality improvement initiative tracking sepsis outcomes (i.e., short-term mortality) and quality measures (i.e.–lactate measurement, time to antibiotics, resuscitative endpoints) for ED patients in the Yale Health System. Inclusion criteria were age greater than 18 years and a diagnosis of severe sepsis or septic shock. In addition, for all included patients, the time between the initial ED serum lactate measurement and the lactate measurement on the floor needed to be 24 hours or less. Patients were excluded from the study if they were discharged to home or were documented as desiring only comfort care measures prior to or during the ED admission.

We based definitions of systemic inflammatory response syndrome (SIRS) criteria, presumed or documented source of infection, end-organ dysfunction, and classification of a patient’s sepsis category upon the model proposed by the International Sepsis Definitions Conference’s consensus statement.^17^ End-organ dysfunction was defined as any one of the following findings: transient systolic blood pressure less than 90 mmHg that responded to fluid resuscitation; lactate level greater than or equal to 2mmol/L; altered mental status from baseline; elevation of any coagulation factor (in the absence of heparin or warfarin therapy); unexplained acidosis marked by an arterial pH less than 7.35 or a serum bicarbonate level less than 21mEq/L; elevation of bilirubin (direct or indirect) from baseline; hypoxemia marked by a pulse oximetry reading less than 90 percent or presence of a significant oxygen requirement; acute kidney injury defined as a creatinine greater than 0.5mg/dL from baseline level or abnormal if no baseline was available; and/or any troponin elevation from baseline.

We collected baseline demographic information for all patients. Data were recorded in a standardized fashion onto a data collection form by medical student investigators under the supervision of a faculty investigator. We entered data from the chart review into an Excel (Microsoft Corp., Redmond, Washington) database. Weekly meetings were conducted to review progress and the data extraction process. Lactate data collected in the database included the time and measured value for the initial ED, peak ED, initial admission, and peak admission serum lactate levels. For data points that were not present in the chart, labs other than serum lactate levels were considered to be normal and were not calculated into the mean value; Glasgow Coma Scale (GCS) scores that were not explicitly recorded were interpreted from the documented neurologic exam or excluded from the mean value if an exam was not documented. There were no missing vital signs or lactate values among our sample. The two chart extractors collected overlapping data points with 95% agreement.

We calculated lactate clearance as a percentage of lactate cleared between the initial ED lactate blood draw and the initial admission lactate level by modeling after previously developed formulas: (Initial ED lactate level - Admission ED lactate level)/Initial ED lactate level^18^,^25^. Patients who had a negative value for lactate clearance based upon this formula were considered to have not cleared lactate; all other patients (i.e., those that had a zero or positive value) comprised the lactate clearance group. The time period over which clearance occurred was calculated using the date and time information for each lab draw. We compared the 28-day in-hospital mortality rate among all-comers who cleared lactate with those who showed an increase in lactate levels at the time of admission.

We also calculated Acute Physiology and Chronic Health Evaluation II (APACHE II) and Mortality in Emergency Department Sepsis (MEDS) scores,^8^,^11^ For MEDS calculations, because “rapidly terminal comorbid disease” data was not explicitly available in the registry, we used patients with cancer receiving chemotherapy as a surrogate of terminal disease. Secondary analyses were performed to determine differences between the lactate clearance group and the patients with negative clearance for variables related to baseline characteristics, disease severity, and ED treatments. In addition, we assessed markers of morbidity outcome between the two groups by comparing rates of vasopressor use, steroid administration, mechanical ventilation, and source control between the two groups.

We completed statistical analysis using SPSS 11.0 (Chicago, IL, USA) and GraphPad (La Jolla, CA, USA). For all categorical variables, relationships were determined using two-tailed Fisher’s exact tests or a chi-squared test. If the data contained continuous variables, independent samples, 2-tailed t-tests were used. Findings were deemed statistically significant for all values of p<0.05. Unless otherwise specified, all reported values in the manuscript, tables, and figures present mean±standard deviation (SD).

## RESULTS

Of the 245 patients in the registry with serial lactate levels obtained both in the ED and on the floor, 207 met inclusion criteria for our study. We excluded 38 patients (15.5%) for having greater than 24 hours between lactate levels in the ED and the floor. The sample consisted of 110 males (53.1%) with a mean age of 63 years±17.9 years. Overall patient characteristics are summarized in Table 1. One hundred thirty-six patients (65.7%) met criteria for severe sepsis and 71 patients (34.3%) met criteria for septic shock. Patients’ mean total number of organ dysfunction signs was 3.63±2.0 with 95 patients (45.9%) having four or more organs with dysfunction.

A summary of ED interventions is described in Table 2. One hundred ninety-four patients (93.7%) in the study received antibiotics in the ED. Fifty-two patients (25.1%) received some form of vasopressor support in the ED and nearly 40% of patients required vasopressor support within 72 hours of admission to the hospital. Eighty-six patients required mechanical ventilation either in the ED or at some point during their hospitalization. One hundred sixty-one patients (77.8%) were admitted to an ICU setting. The overall 28-day in-hospital mortality rate for our cohort was 19% (39 patients).

The mean initial ED lactate in the clearance group was 3.2±2.1mmol/L and the mean for the non-clearance group was 3.1±3.7mmol/L (95% CI, [−1.2 to 1.4]; p=0.861). In contrast, admission lactate levels differed significantly between the non-clearance group’s mean level of 4.7±4.8 mmol/L and the clearance group’s mean lactate of 1.7±1.2 (95 % CI, [−4.6 to −1.4]; p<0.001). Table 3 compares demographical and clinical variables between the clearance and non-clearance groups.

**Figure 1:**
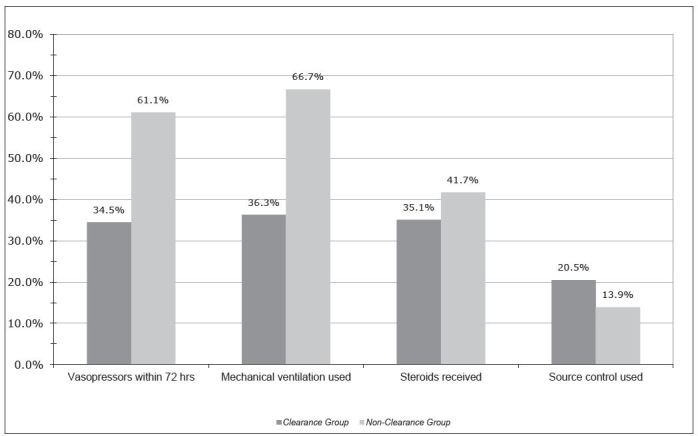
Hospital interventions for clearance and non-clearance groups. A significantly higher ratio of patients in the non-clearance group required vasopressor support within 72 hours of admission compared with the rate of vasopressor use in the clearance group (p<0.01). A greater percentage of patients in the non-clearance group required mechanical ventilation for any point during the hospitalization (p=0.001). There were no significant differences in the rates of steroid use or source control between clearance and non-clearance groups.

The percentages of patients receiving vasopressor support in the ED were 24% (n=41) and 30.6% (n=11) for the clearance and non-clearance groups, respectively (p=0.405). As shown in Figure 1, the non-clearance group had a rate of overall hospital vasopressor use of 61.1% (n=22) whereas only 36.8% (n=63) in the clearance group received vasopressors after admission (p=0.009). There was also a difference in the rate of hospital use of mechanical ventilation between the lactate clearance group and the patients who did not clear lactate (36.3% (n= 62) vs. 66.7% (n=24), p=0.001). Rates of corticosteroids and source control procedures were similar among the two groups. The mean ED length of stay for the clearance group was 6.5±3.33 hours contrasted to 5.98±3.93 hours for the Non-clearance group (p=0.41).

In hospital 28-day mortality rates were 12.7% (8 of 63 patients) for patients who had initial lactates less than 2.0mmol/L, compared with mortality rates of 19.5% (17 of 87 patients) among patients with an initial lactate between 2.0 and 4mmol/L and 24.6% (14 of 57 patients) for those with lactates greater than 4.0mmol/L (p=0.246).

**Figure 2:**
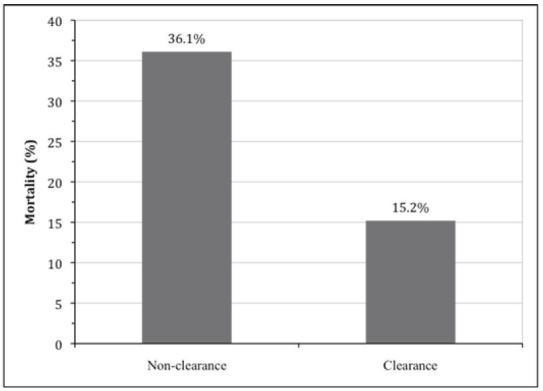
Mortality rates by lactate clearance group. 28-day in-hospital mortality rates were significantly lower among patients who cleared lactate (15.2% mortality) compared with those who did not (36.1% mortality, p<0.01).

For our cohort, the mean time between the initial ED lactate measurement and the second-floor lactate blood draw was 9 hours and 8 minutes±4 hours and 46 minutes. As shown in Figure 2, the mortality rate was 36.1% (13 of 36 patients) among those who did not clear their lactate level after admission compared with the mortality rate of 15.2% (26 of 171 patients) for those in the lactate clearance group (p=0.008). Further, among the subgroup of 144 patients with an initial ED lactate of 2 mmol/L or higher, 28-day mortality rates were 62.5% for the non-clearance group (10 of 16 patients) compared with 16.4% (21 of 128 patients) in the clearance group (p<0.001).

## DISCUSSION

We have shown in a real-world cross-sectional study that 28-day in-hospital mortality rates are significantly higher among patients who have no lactate clearance upon admission to the hospital compared with those who have clearance. Our findings complement a growing body of literature, consisting of both retrospective studies and prospective and randomized clinical trials that demonstrate the non-invasive variable of lactate clearance can be used to predict 28-day mortality among patients in the ED with severe sepsis or septic shock.^18^,^25^,^30^,^31^,^32^

Bakker et al. found that among the septic shock population, a shorter “lac-time” (defined as the total duration of elevated blood lactate levels) could predict survivability but also could predict lower organ failure scores, lending credence to the emerging concept of serial lactate measurement.^15^ Specific application to the ED setting came with Nguyen’s novel use of the formula for lactate clearance during the first six hours of care, and these authors showed a statistically and clinically significant difference in outcome.^18^ They found that although there is often no statistical difference among patients’ initial lactate levels, those patients who were unable to improve their lactic acidosis were more likely to develop organ failure and had higher 24-hour and 60-day mortality rates.^15^,^18^ Nguyen et al. did not find a significant difference in the use of ED vasopressors or fluid among survivors and non-survivors in their sample, and our cohort similarly revealed no differences between clearance and non-clearance groups for ED vasopressor usage. In addition, we found no significant difference in antibiotic administration rates between the two groups. In an analysis using data from prospectively collected registries from three urban hospitals, Arnold et al., showed similar results to Nguyen’s work, suggesting that lactate clearance of 10% or greater from initial values was associated with significant mortality benefits.^25^ Our study shows comparable results using a slightly different definition of “clearance” in that we examined a binary distinction (i.e., decrease or no change in lactate versus any increase in lactate), which we feel can be easily calculated by the clinician.

Our results are also similar to work from other arenas, including the trauma literature, that supports the notion of lactate clearance as a marker for ongoing tissue hypoxia and a predictor of mortality. The use of normalized lactate clearance has been associated with improved outcomes in several critical illness settings, including both trauma patients^33^,^34^ and patients with circulatory arrest.^35^–^37^ Our findings also show a significantly higher requirement for hospital vasopressors within 72 hours of admission for the non-clearance group and further revealed a significantly higher rate of mechanical ventilation among the non-clearance group, a finding that was not seen in the prior work by Nguyen et al. but has been demonstrated in a recent study among trauma patients in which impaired 24-hour lactate clearance increased the likelihood of requiring mechanical ventilation.^38^

Among our sample, fewer patients in the non-clearance group received fluids compared with those patients who had clearance of lactate suggesting that under-resuscitation with intravenous fluids, independent of receipt of antibiotics, may correlate with impaired lactate clearance; the patients in the non-clearance group likely had sustained global tissue hypoxia due to ongoing physiologic derangements. Therefore, it appears that patients who are not clearing lactate are sicker in spite of having similar severity of illness scores to the control group. This could be because a proportion of patients didn’t receive adequate ED fluid resuscitation, or it could illustrate that lactate is a better prognostic variable for the ED setting. Furthermore, extrapolation from our results suggests that lactate clearance might be a useful endpoint for ED resuscitation, but further investigation will undoubtedly be needed to assess its exact potential.

Currently, lactate clearance is not readily identified as a variable that can be used to determine the therapeutic endpoint for patients with sepsis.^23^ However, a recent study has shown lactate clearance may be used as a surrogate for invasive central venous oxygen saturation when implementing resuscitative strategies.^26^ If lactate clearance continues to be associated with improved outcome, as in our study and other recent work,^18^,^25^,^26^ it may suggest that normalized lactate values can be used as a therapeutic endpoint among this patient population, should be incorporated into sepsis bundles, and might dictate safe disposition from the ED or ICU to a general medical floor.

## LIMITATIONS

Limitations of our study include its retrospective extraction from a prospective registry and chart abstractors were not blinded to the study hypothesis. Several data points were unavailable in the sepsis registry, and calculation of GCS, for example, relied upon use of documented neurologic status at triage or during the physical exam. Despite this limitation, our cohort included 207 patients, which is among the highest in any study of lactate clearance and ED sepsis to date, and we had strong agreement between the two investigators recording overlapping data points. The study was also prone to a length bias as there was not a specific protocol guiding the intervals lactate levels were drawn (i.e., time to initial and time to repeat lactate level) —this was left at the discretion of the treating teams and could have been delayed for various reasons, thus confounding results. However, there is still potential merit in assessment at variable time-periods. What is perceived as “lactate clearance” may also be interpreted, given the short duration of elevation seen in other patient populations (i.e., grand mal seizure patients), as a decrease in lactate production.^39^ This interruption in lactate production likely ensues from resuscitative procedures reversing global tissue hypoxia and oxygen delivery and consumption mismatches. It is feasible that the termination of lactate production reflected by lactate clearance formulas may perhaps better confer prognostic information in the early (<24 hours) phase of ED and inpatient care. Despite these considerations that merit further investigation, in our study the mean time over which lactate clearance was measured was only three hours greater than the study by Nguyen et al. and there was no significant difference in the mean time over which lactate clearance was calculated between our clearance and non-clearance groups.

Similarly, our results could be affected by selection bias, in that sicker patients may have been prone to getting lactate levels checked more frequently. However, we found no significant differences between the clearance and non-clearance group for initial lactate level and also found no significant difference in APACHE II score between the two groups, suggesting that the overall population had similar disease severity at the time of presentation and initial lactate draw. However, the proportion of patients who received antibiotics or IVF in the ED was higher in the clearance group (p=0.002 for IVF, p=0.054 for antibiotic administration). This confounder could account for the observed differences in mortality, and perhaps underscores the importance of administering these key interventions in a time-sensitive fashion in the ED.

Additionally, selection bias may have been why some patients were excluded because an initial lactate was not measured, but this group was small. Our overall sample population had even distribution by gender, had a mean age of 63 years which is similar to the mean age of the sample used by Nguyen et al., and also had similar characteristics between the clearance and non-clearance groups for initial laboratory parameters, SIRS presentations, ED length of stay, and lactate levels. Thus we feel our “real world” findings can be compared with those of prior studies and are applicable to the broader population of patients presenting to the ED with severe sepsis or septic shock.

## CONCLUSION

Based on our findings, we conclude that lactate clearance appears to correlate with short-term survival among patients with severe sepsis or septic shock. Lactate clearance could serve as an efficient tool for mortality risk-stratification similar to more complex scoring systems and could potentially provide critical information about response to treatment. Despite the need for further prospective validation studies, this study reveals that the ability to clear lactate or halt lactate production could have potential as a predictor of mortality among patients presenting to the ED with severe sepsis or septic shock, in aiding with disposition, or in recognizing patients who require additional resuscitation.

## Figures and Tables

**Table 1:** Patient characteristics (n=207) in study of lactate clearance as predictor of survival in patients with severe sepsis.

Patient characteristics	n (%)
Male	110 (53.1%)
Mean age ± SD (years)	63.17 ± 17.9
Diagnosis	
Severe sepsis	136 (65.7%)
Septic shock	71 (34.3%)
Diagnostic criteria	
Mean number of SIRS criteria ± SD	2.99 ± 0.77
Documented source of infection	
Genito-urinary	24 (11.6%)
Intra-abdominal	27 (13.0%)
Pneumonia	54 (26.1%)
Soft tissue	16 (7.7%)
Other†	19 (9.2%)
Mean number of organ dysfunctions ± SD	3.63 ± 2.0
Transient hypotension	86 (41.5%)
Lactate level ≥2mmol/L	144 (69.6%)
Unexplained acidosis	97 (46.9%)
Altered mental status	72 (34.8%)
Low platelet count	31 (15.0%)
Elevated bilirubin level	81 (39.1%)
Coagulopathy(without prior anticoagulation)	31 (15.0%)
Acute renal failure	98 (47.3%)
Hypoxemia	64 (30.9%)
Troponin elevation	52 (25.1%)
Past medical history	
Alcohol abuse	26 (12.6%)
Asthma	10 (4.8%)
Cancer	54 (26.1%)
Cancer with chemotherapy	22 (10.6%)
Congestive heart failure	45 (21.7%)
Coronary artery disease	47 (22.7%)
Chronic altered mental status	25 (12.1%)
Chronic obstructive pulmonary disease	38 (18.4%)
CVA/transient ischemic attack	29 (14.0%)
Diabetes	74 (35.7%)
End stage renal disease	25 (12.1%)
HIV or HIV/AIDS	9 (4.3%)
Hypertension	108 (52.2%)
Immunosuppression	22 (10.6%)
Liver disease	15 (7.2%)
Residing in extended care acility	59 (28.5%)
Mean MEDS score ± SD§	9.05 ± 4.09

*SIRS,* systemic inflammatory response syndrome; *CVA*, cerebrovascular accident; *HIV*, human immunodeficiency virus; *AIDS*, acquired immune deficiency syndrome

† e.g., central nervous system infection or line infection.

*MEDS,* mortality in emergency department sepsis

§ N = 206.

**Table 2:** Interventions and treatment (n=207).

	n (%)
Mean length of ED stay ± SD (hours:minutes)	6:25 ± 3:26
Mean intravenous fluid amount ± SD (L)	3.43 ± 2.33
Antibiotic treatment	
Received	194 (93.7%)
Mean time ± SD (hours:minutes)	2:34 ± 2:12
Type of antibiotic†	
Acyclovir	4 (2.1%)
Ampicllin	4 (2.1%)
Ceftazadime	8 (4.1%)
Ceftriaxone	45 (23.2%)
Ciprofloxacin	45 (23.2%)
Doxycycline	33 (17.0%)
Metronidazole	22 (11.3%)
Gentamicin	8 (4.1%)
Ampicillin/sulbactam	8 (4.1%)
Vancomycin	122 (62.9%)
Piperacillin/tazobactam	112 (57.4%)
Other§	9 (4.6%)
Cultures	
Blood culture drawn	199 (96.1%)
Urine culture drawn	139 (67.1%)
Other culture drawn‡	63 (30.4%)
Any culture positiveΔ	140 (34.9%)
Hospital vasopressors	
Less than 72 hours after admission	81 (39.1%)
Greater than 72 hours after admission	7 (3.4%)
Hospital use of dobutamine	15 (7.2%)
Hospital use of corticosteroids	75 (36.2%)
Source control†	40 (19.3%)
Hospital use of mechanical ventilation	86 (41.5%)

*ED,* emergency department

† N=194 (of those patients receiving antibiotics).

§ amoxicillin, clindamycin, meropenem, moxifloxacin, trimethoprim-sulfamethoxazole.

‡ e.g., sputum, wound, or cerebrospinal fluid cultures.

Δ N=401 (of those cultures drawn).

† abscess drained, line pulled, endoscopic or operative management.

**Table 3:** Baseline characteristics and therapies for clearance and non-clearance groups.

Variable	Clearance group (N=171)	Non-clearance group (N=36)	p-value
Age (years)	63.3 ± 18.0	62.53 ± 17.52	0.814
Diagnostic variables			
Severe sepsis (%)	67.3	58.3	0.337
Number of SIRS criteria	3.03 ± 0.75	2.78 ± 0.80	0.073
Source of infection (%)			
Genito-urinary	11.1	13.9	0.577
Intra-abdominal	14.0	8.3	0.429
Pneumonia	25.7	27.8	0.835
Soft tissue	7.6	8.3	1.00
Other†	9.4	8.3	1.00
Number of organ dysfunctions	3.54 ± 1.87	4.03 ± 2.50	0.187
Past medical history (%)			
Alcohol abuse	13.5	8.3	0.581
Cancer	26.9	22.2	0.678
Cancer with chemotherapy	8.8	19.4	0.074
Congestive heart failure	19.3	33.3	0.076
Coronary artery disease	23.4	19.4	0.669
Chronic obstructive pulmonary disease	15.2	33.3	0.017^a^
Diabetes	37.4	27.8	0.340
End stage renal disease	11.7	13.9	0.778
Hypertension	49.7	63.9	0.144
Immunosuppression	9.9	13.9	0.550
Liver disease	7.0	8.3	0.729
Residing in extended care facility (%)	26.9	36.1	0.310
Glasgow coma scale scoreψ	13.6 ± 2.8	13.2 ± 3.5	0.431
MEDS score	8.78 ± 3.96	10.40 ± 4.48	0.032^a^
APACHE II score	18.6 ± 7.0	21.1 ± 8.6	0.069
Laboratory values			
WBC (per mm^3^)‡	13.99 ± 8.10	16.71 ± 18.57	0.168
Hematocrit (%)‡	37.72 ± 7.41	35.60 ± 8.01	0.130
Platelet count (per mm^3^)‡	260.8 ± 124.4	222.1 ± 129.8	0.097
Creatinine (mg/mL)	2.4 ± 2.1	2.4 ± 1.9	0.942
Blood cultures drawn (%)	97.1	91.7	0.145
Urine cultures drawn (%)	70.2	52.8	0.052
Initial ED lactate (mmol/L)	3.26 ± 2.14	3.14 ± 3.73	0.804
Initial admission lactate (mmol/L)	1.76 ± 1.25	4.76 ± 4.81	<0.001^a^
Therapy (%)			
Intravenous fluid administered in ED	98.8	86.1	0.002^a^
Amount of intravenous fluid (L)Δ	3.41 ± 2.26	3.57 ± 2.74	0.720
Antibiotics administered in ED	95.3	86.1	0.054
Central line placement in ED	45.0	61.1	0.099
ED vasopressor use	24.0	30.6	0.405

*SIRS,* systemic inflammatory response syndrome; *MEDS,* mortality in emergency department sepsis; *APACHE II,* Acute Physiology and Chronic Health Evaluation II; *WBC*, white blood cell; *ED,* emergency department

† e.g., line infection or central nervous system infection.

ψ N=165 for clearance group.

‡ N=35 for non-clearance group.

Δ N=169 for clearance group.

N=34 for non-clearance group

*ED,* emergency department

a Statistically significant, p<0.05.
